# Diseases of Nervous System

**Published:** 1905-09-23

**Authors:** 


### DISEASES OF NERVOUS SYSTEM.

**On the Administration of Iodide of Potassium.**—Iodide of potassium should always be given in solution, well diluted, and, if possible, not on an empty stomach. The drug may simply be dissolved in water, but a far better plan is to dissolve it in milk, for this not only disguises the taste but also in some degree prevents any disagreeable after-effects of the drug. The iodide, in watery solution, may be added to a wineglass or more of milk. The drug should be perfectly pure, containing no free iodine. The patient should be told to observe strict cleanliness of the skin by daily baths, for iodide eruptions are due to decomposition of the salt excreted by the fatty acids of the perspiration. While the patient is taking iodide calomel should not be dusted into the eye, else a strong caustic effect is produced. The drug is of great use in many conditions, and the following practical observations may be of use. It should not be given in primary syphilis, nor in the secondary stage until the patient has had at least six months of treatment with mercury. In the simple secondary lesions it is useless, but may prove beneficial in recurrences. Its great use in syphilis is in tertiary affections, where it should be given in large doses. And in such cases, especially when some vital organ is affected, toxic symptoms such as rhinitis or pustulation of the skin, should be disregarded; these may disappear as the dose is increased, or even if they do not it is all-important to saturate the patient with the drug. If no good results after two months' trial it should be discontinued. In parasyphilitic affections such as locomotor ataxy and general paralysis, iodide is quite useless. Chronic endarteritis, whether syphilitic or not, is greatly improved by iodide, which should be given in small doses over a long period; and similarly in chronic endocarditis. Cases of asthma must be carefully selected, for in some iodide does harm; if the attacks are due to nasal polypi or other reflex nasal causes, iodide makes matters worse; on the other hand, if they are caused by tonic spasm of the bronchioles, excellent results are obtained. Chronic bronchitis, chronic nephritis, and lead poisoning are often greatly benefited by iodide. The drug is useless in acute and also in most cases of muscular rheumatism; in some cases of the muscular form it does good when combined with gelsemium. Gout, unless associated with rheumatism, is not favourably affected by iodide.

**Treatment of General Paralysis by Urotropin.**—Two remarkable cases of apparent amelioration of general paralysis of the insane by urotropin have recently been recorded by MacHardy. One had suffered from symptoms of the disease for two years, and was dangerous and violent, with grandiose delusions. Paralysis of the bladder supervened, and a catheter had to be used. He was then given urotropin, beginning with 5 grains daily and increasing to 15 grains. In less than seven months all the symptoms of general paralysis had disappeared; he was sent home on probation, and was well some months later. The other case was very similar to the above. Two cases of a disease liable to remissions, such as general paralysis, are not sufficient to build much upon. But considering the fact that Ford Robertson and others believe that bacterial toxins generated in the body play a great part in the disease, the above-mentioned cases suggest that urotropin should be given a more extensive trial. The drug is free from risk in moderate doses and is readily taken.

[1] Med. Rec., April 1. [2] Brit. Med. Jour., vol. i., 1905, p. 185.

